# Supplementary material for: The genome sequence of a long-legged fly, Argyra leucocephala (Meigen, 1824)
Source: Wellcome Open Res. 2025 Jun 4;10:309. [Version 1] doi: 10.12688/wellcomeopenres.24307.1 (PMC12355172; doi:10.12688/wellcomeopenres.24307.1)
Supplement: Peer review history

# Reviewer report: Reviewer response for version 1

**DOI:** 10.21956/wellcomeopenres.26807.r125885

**Published:** 2025-08-14

**Authors:** Arun Arumugaperumal

**Affiliations:** 1 Rajalakshmi Engineering College, Chennai, Tamil Nadu, India

The authors present the genome sequence of *Argyra leucocephala*, a long-legged fly. The photograph presented in figure 1 is clear. However, a photo of the alive fly besides the specimen may improve the understanding of readers. The assembly reported here is of size 917.38 Mb spread across 6 chromosomes and a mitogenome of size 18.95 kb. The annotation through standard pipeline has identified 22,809 proteins. This indicates the high quality of the genome assembly. Till this time any other Argyra genome is not available. This is the first one to be published. Hence a BUSCO score of 93% also seems valid. The data note can be indexed. The genome sequence reported herewith will be a valuable resource to study the biology of the organism.

Are sufficient details of methods and materials provided to allow replication by others?

Yes

Is the rationale for creating the dataset(s) clearly described?

Yes

Are the datasets clearly presented in a useable and accessible format?

Yes

Are the protocols appropriate and is the work technically sound?

Yes

Reviewer Expertise:

Bioinformatics; Genomics

I confirm that I have read this submission and believe that I have an appropriate level of expertise to confirm that it is of an acceptable scientific standard.

# Reviewer report: Reviewer response for version 1

**DOI:** 10.21956/wellcomeopenres.26807.r125890

**Published:** 2025-07-04

**Authors:** Komal Kumar Bollepogu Raja

**Affiliations:** 1 Baylor College of Medicine, Houston, Texas, USA

The article by Falk S. and Crowley L.M. presents the genome sequence of *Argyra* *leucocephala* with a total length of 917.38 megabases. The genome assembly appears to be of very high quality. The number of contigs is very low and the longest contig is 212.71 Mb suggesting contiguous assembly. The contig assembly metrics are very impressive. The BUSCO score and k-mer completeness are impressive. Further, the percentage mapped to chromosomes is very high. The experimental methods are robust and allow for reproducibility. The Hi-C map is high-quality and represents well-assembled genome. Overall, this is a very high quality genome and an excellent resource for genomics research.

Are sufficient details of methods and materials provided to allow replication by others?

Yes

Is the rationale for creating the dataset(s) clearly described?

Yes

Are the datasets clearly presented in a useable and accessible format?

Yes

Are the protocols appropriate and is the work technically sound?

Yes

Reviewer Expertise:

genomics, bioinformatics, single cell genomics, neurobiology

I confirm that I have read this submission and believe that I have an appropriate level of expertise to confirm that it is of an acceptable scientific standard.

# Reviewer report: Reviewer response for version 1

**DOI:** 10.21956/wellcomeopenres.26807.r125891

**Published:** 2025-06-25

**Authors:** Henrique Antoniolli

**Affiliations:** 1 Universidade Federal do Rio Grande do Sul (UFRGS), Porto Alegre, Brazil

The present study reports a highly contiguous genome assembly for *Argyra leucocephala*, a long-legged fly. The applied methodology is well described and the produced data are available for replication by other researchers. Assembly statistics and gene annotation reached satisfactory results for publishing the genome report.

 However, the Background section did not clearly mention the reasons for sequencing its genome.  Also, this section would benefit from a more detailed paragraph describing the general biology of this species. Therefore, I do not recommend indexing this paper in the current form.

Are sufficient details of methods and materials provided to allow replication by others?

Yes

Is the rationale for creating the dataset(s) clearly described?

Partly

Are the datasets clearly presented in a useable and accessible format?

Yes

Are the protocols appropriate and is the work technically sound?

Yes

Reviewer Expertise:

Genomics, evolution

I confirm that I have read this submission and believe that I have an appropriate level of expertise to state that I do not consider it to be of an acceptable scientific standard, for reasons outlined above.
